# Limb lengthening and deformity correction with externally controlled motorized intramedullary nails: evaluation of 50 consecutive lengthenings

**DOI:** 10.1080/17453674.2018.1534321

**Published:** 2018-10-29

**Authors:** Joachim Horn, Ivan Hvid, Stefan Huhnstock, Anne B Breen, Harald Steen

**Affiliations:** a Section of Children’s Orthopaedics and Reconstructive Surgery, Division of Orthopaedic Surgery, Oslo University Hospital;;; b Biomechanics Lab, Division of Orthopaedic Surgery, Oslo University Hospital, Norway

## Abstract

Background and purpose — Limb lengthening with an intramedullary motorized nail is a relatively new method. We investigated if lengthening nails are reliable constructs for limb lengthening and deformity correction in the femur and the tibia.

Patients and methods — 50 lengthenings (34 Precice and 16 Fitbone devices) in 47 patients (mean age 23 years [11–61]) with ≥12 months follow-up are included in this study. 30 lengthenings were done due to congenital and 20 because of posttraumatic deformity (21 antegrade femora, 23 retrograde femora, 6 tibiae). Initial deformities included a mean shortening of 42 mm (25–90). In 15 patients, simultaneous axial correction was done using the retrograde nailing technique.

Results — The planned amount of lengthening was achieved in all but 2 patients. 5 patients who underwent simultaneous axial correction showed minor residual deformity; unintentionally induced minor deformities were found in the frontal and sagittal plane. The consolidation index was 1.2 months/cm (0.6–2.5) in the femur and 2.5 months/cm (1.6–4.0) in the tibia. 2 femoral fractures occurred in retrograde femoral lengthenings after consolidation due to substantial trauma. There were 8 complications, all of which were correctable by surgery, with no permanent sequelae.

Interpretation — Controlled acute axial correction of angular deformities and limb lengthening can be achieved by a motorized intramedullary nail. A thorough preoperative planning and intraoperative control of alignment are required to avoid residual and unintentionally induced deformity. In the femur relatively fast consolidation could be observed, whereas healing was slower in the tibia.

Distraction osteogenesis by use of an external fixator is a well-established method. To overcome problems associated with the use of external fixation, several techniques that allow early removal of the frame have been developed, including lengthening over a nail (Bost and Larsen 1956, Paley et al. 1997), lengthening and then nailing (Faber et al. 1991, Rozbruch et al. 2008), and lengthening and then plating (Harbacheuski et al. 2012). Further progress has been made by the development of mechanical (Guichet 1999, Cole et al. 2001) and externally controlled motorized intramedullary lengthening devices like the Fitbone nail (Betz et al. 1990, Baumgart et al. 1997) and the Precice nail (Kirane et al. 2014, Schiedel et al. 2014, Paley 2015).

Lengthening with a fully implantable motorized and remote-controlled intramedullary nail is a relatively new method, and only a limited number of reports exist evaluating these lengthening devices. Hence we evaluated our first 50 consecutive cases of limb lengthening with motorized nails in terms of: (1) achieved lengthening and alignment, (2) unintentionally induced deformity, (3) healing of the regenerate and (4) complications. Furthermore, we compared the antegrade nailing technique in the femur with the retrograde nailing technique with respect to these outcome measures.

## Patients and methods

### Patients

50 lengthenings in 47 patients (24 men) with a follow-up of at least 12 months after consolidation of the regenerate are included in this study. 15 of these 50 lengthenings have been previously published (Horn et al. 2015) but are also included in the current paper with additional outcome measures added.

The patients’ mean age at operation was 23 years (11–61). The leg length discrepancy (LLD) was caused by various etiologies (Table 1). In 3 patients consecutive lengthening of both femora was performed due to short stature below 2 SD from average adult height (diagnoses: Léri–Weill dyschondrosteosis, achondroplasia, neonatal growth restraint due to prematurity). Initial deformities included shortening in all patients with a mean of 41 mm (25–88). 23 patients received retrograde femoral nails (RFN), 21 antegrade femoral nails (AFN), and 6 tibia nails (TN). None of the patients had been previously lengthened in the respective segment. In 15 procedures, simultaneous axial correction was done using the RFN. 8 of these patients had initial valgus deformity with a mean lateral (positive) mechanical axis deviation (MAD) of 21 mm (4–50), 5 patients had a varus deformity with a mean medial (negative) MAD of –31 mm (–14 to –58) and 2 patients had a femoral procurvatum deformity of 12° and 26°, respectively. In the remaining 35 procedures, isolated lengthening was performed (21 AFN, 8 RFN, and 6 TN). Mean follow-up after consolidation of the regenerate was 28 months (12–72).

**Table 1.** Etiology of LLD and treatment methods

	Total number	Retrograde femoral nail	Antegrade femoral nail	Tibial nail
Etiology	n = 50	n = 23	n = 21	n = 6
Posttraumatic	13	11	1	1
Congenital femoral deficiency/fibula hemimelia	10	5	4	1
Hypoplasia	5	1	4	
Idiopathic	3	2	1	
Pes equino varus	3	1		2
Achondroplasia	2		2	
Léri–Weill syndrome	2		2	
Short stature	2	2		
Beta thalassemia	2		1	1
Hip dysplasia/Perthes	2		2	
Multiple osteochondroma	1	1		
Polio	1		1	
Amniotic band syndrome	1			1
Metaphyseal dysplasia	1		1	
Osteopathia striata	1		1	
Cerebral paresis	1		1	

### Implants

In 34 lengthenings we used the Precice lengthening nail (NuVasive Inc, San Diego, CA, USA) and in 16 lengthenings the Fitbone device (Wittenstein intens GmbH, Igersheim, Germany), both fully implantable motorized lengthening nails.

### Preoperative assessment and surgical technique

All patients were assessed preoperatively with physical examination and calibrated radiographs, including long standing radiographs.

Of 44 femoral lengthenings 21 were done with an AFN (all Precice) and 23 with an RFN (16 Fitbone, 7 Precice). All AFN had a 10° proximal bend and we used a trochanteric entry point for insertion. In 15 RFN acute axial correction was performed when the nail was inserted. All 6 tibial lengthenings were done by use of TN with a 10° bend (all Precice).

For the retrograde technique preoperative planning was done based on the reverse planning method (Baumgart 2009). A 3 mm diameter threaded Steinmann pin was inserted into the proximal and distal fragment to control rotation, followed by performing multiple drill holes at the intended osteotomy site to vent the canal during reaming. This was done to reduce the risk for fat embolism and to potentially enhance the healing of the regenerate by accumulation of reamer debris at the osteotomy site. In the retrograde technique reaming was done in a preplanned way using rigid, straight reamers, and the use of blocking screws to guide the reamers intraoperatively and to maintain the acutely performed deformity correction. In antegrade femoral and in tibial nailing flexible reamers over a ball-tipped guide wire were used. After reaming, osteotomies were completed in a percutaneous manner (10 mm skin incision) with fan-shaped drilling using a 4-mm diameter drill bit and a 10 mm osteotome.

Prophylactic antibiotics were given every 90th minute during surgery.

### Postoperative care and follow-up

Weight-bearing up to 20 kg was permitted immediately. Dalteparin was given 6 hours postsurgery (2500 units) and once a day for the first 7 days after surgery (2500–5000 units depending on body weight). Lengthening in the femur was initiated 7 days after surgery with a distraction rate of 1 mm/day (3 times 0.33 mm/day) and in the tibia 10–14 days after surgery with a rate of 0.66 mm/day (2 times 0.33 mm). Patients were followed with outpatient visits every 2nd week during lengthening and every 6th week during the consolidation phase. They had physical therapy 3 times per week besides daily home exercises with the main emphasis on active and passive extension of the knee. Patients with congenitally short femur and insufficient cruciate ligaments were obliged to use a knee orthosis that kept the knee in full extension for 22 hours per day during the distraction phase and for the first 2 months after completed lengthening.

Full weight-bearing was permitted when radiographs showed at least 3 consolidated cortices of the regenerate.

### Outcome parameters

The outcome parameters were: (1) achieved lengthening and alignment, (2) unintentionally induced malalignment, (3) healing of the regenerate and (4) complications. Long standing radiographs were obtained from all patients preoperatively, and after consolidation of the regenerate. Deformity analysis was done based on the malalignment test and malorientation test as described by Paley (2002).

Analysis of alignment included evaluation of any unintentionally induced malalignment, which might occur due to lengthening along the anatomical axis in the femur resulting in a shift of the mechanical axis (MA) to lateral (Burghardt et al. 2012) or due to unintended angulation of bone fragments (Muthusamy et al. 2016). For this purpose, mechanical axis deviation (MAD) and mechanical lateral distal femoral angle (mLDFA) values were measured in all isolated femoral lengthenings preoperatively and after lengthening, as well as medial neck shaft angle (MNSA), which was measured in all antegrade nails. Angulation in the sagittal plane in the femur was measured on standard lateral femur radiographs. In the tibia medial proximal tibia angle (MPTA) and posterior proximal tibia angle (PPTA) were measured preoperatively and after consolidation of the regenerate. Furthermore, the precision of the MAD analyses was calculated by evaluating repeated measurements of the healthy lower extremity in 28 of the individuals included in this study.

Radiographs of the bone segment under lengthening were evaluated with respect to the bone regenerate and the amount of achieved lengthening. Consolidation index was defined as the time from the osteotomy to radiographic consolidation divided by the achieved lengthening distance in centimeters. Complications were graded into problems, obstacles, and sequelae according to Paley (1990).

### Statistics

Statistical analyses were done based on Student’s t-test and the paired t-test. P-values less than 0.05 were considered statistically significant. In order to comply with requirements for independent observations, the 2nd lengthening in 3 patients with bilateral lengthening was excluded from all statistical analysis.

### Ethics, funding, and potential conflicts of interest

The study was approved as a quality control study by the local office of privacy protection and information safety (Ref. 2016/11785). No funding was received and there are no competing interests declared

## Results

### Patients

The patient’s mean age at surgery was 23 years (11–61) (20 patients were ≤20 years of age). Mean age in the group with AFN was 20 years (11–52) and 27 years (15–61) in RFN group. The age distribution in these groups was similar. Patients who received TN were 21 years (16–30). All patients who received either RFN or TN were skeletally mature.

### Surgical procedure

There were no intraoperative complications, and none of the patients had any significant blood loss intra- or postoperatively. Mean duration of all surgeries was 159 min (65–330). Mean duration of surgery for the AFN was 112 min (65–163) and 187 min (120–330) for the RFN (p = 0.005). In TN the mean surgical time was 170 min (147–184).

### Achieved lengthening and alignment

A mean lengthening of 40 mm (25–65) was achieved (Table 2). The planned amount of lengthening was accomplished in all but 2 patients in whom femoral lengthening was terminated due to knee pain and loss of some knee extension when 40 and 50 mm of lengthening had been achieved. No loss of length was found during further follow-up in any patient. MAD (medial deviation = negative, lateral deviation = positive) in all procedures with simultaneous axial correction was significantly reduced and changed from mean 23 mm (–58 to 26) preoperatively to 6 mm (–23 to 24) postoperatively (p = 0.01). Preoperative mLDFA changed from mean 85° (73–102) to 88° (80–91°) (p < 0.01). 5 patients who underwent simultaneous axial correction showed some residual deformity. 4 of these patients had a varus deformity with a mean value of 17 mm (12–23) MAD; 1 patient had residual valgus deformity with 24 mm MAD.

**Table 2.** Outcome measures (mean and range values) of achieved lengthening and alignment, including unintentionally induced malalignment

	Preoperative	Postoperative	Change	p-value
Achieved lengthening in all procedures (n = 50)				
Lengthening (mm)	Intended	Achieved		
	41 (25–80)	40 (25–65)		
Achieved alignment in cases with simultaneous axial correction (n = 15)				
MAD (mm)	23 (–58 to 26)	6 (–23 to 24)	17 (4 to 58)	0.01
mLDFA (°)	85 (73 to 102)	88 (80 to 91)	5 (2 to 13)	< 0.01
Unintentionally induced malalignment in cases of isolated lengthening (n = 35)				
Frontal plane alignment femur				
MAD (mm) (antegrade and retrograde nail)	1 (–20 to 43)	0 (–17 to 32)	3 (0 to 11)	0.9
MNSA (°) (antegrade nail)	124 (106 to 138)	122 (107 to 135)	–3 (–9 to 3)	0.008
mLDFA (°) (retrograde nail)	90 (85 to 95)	90 (87 to 94)	2 (0 to 4)	0.6
Sagittal plane alignment femur				
Antegrade femoral nail (°)	7 (0 to 11)	5 (0 to 10)	–2 (–9 to 4)	0.02
Retrograde femoral nail (°)	6 (0 to 16)	4 (0 to 12)	–2 (–9 to 7)	0.04
Frontal and sagittal plane tibia				
MPTA (°)	87 (85 to 89)	88 (86 to 89)	1 (1 to 2)	0.2
PPTA (°)	79 (75 to 81)	76 (75 to 81)	3 (0 to 9)	0.1

MAD: mechanical axis deviation, medial MAD (–), lateral MAD (+);

mLDFA: mechanical lateral distal femoral angle;

MNSA: medial neck shaft angle;

MPTA: medial proximal tibia angle;

PPTA posterior proximal tibia angle;

sagittal plane: recurvatum(–), procurvatum (+).

### Unintentionally induced malalignment

#### Frontal plane femur

A change of MA in isolated femoral lengthening (n = 29) was not intended. Mean MAD preoperatively was 1 mm (–20 to 43) and postoperatively MAD was 1 mm (–17 to 32). The change of MAD from preoperatively to when lengthening was completed was on average 3 mm (0–11), a finding that was not statistically significant (p = 0.9) (Table 2). Change of MNSA would allow for quantifying unintended malalignment in the proximal femur when using antegrade femoral lengthening nails. In the group with AFN preoperative MNSA was mean 124° (106–138) and postoperatively 122° (107–135); this difference is statistically significant (p = 0.008) but not clinically relevant. In 8 patients a varization of the proximal fragment of ≥5° was observed, resulting in a shift of the mechanical axis 10 mm to the medial side in 1 patient. In those patients who underwent isolated lengthening (n = 8) or lengthening combined with sagittal plane deformity correction (n = 2) by means of a RFN, changes in mLDFA would allow for conclusions regarding unintentionally induced frontal plane malalignment. However, it has to be noted that isolated lengthening along the mechanical axis would normally lower the mLDFA. Preoperative mLDFA was mean 89° (85–95) and postoperatively 90° (86–94).

#### Sagittal plane femur

In the sagittal plane recurvatum (–) and procurvatum (+) of fragments were measured, showing a mean change of –2° (–9 to 4) for the AFN, and –2° (–9 to 7) for RFN. This means a tendency towards dorsiflexion of the fragments (Table 2), which has to be considered a result of reduction of the natural femoral procurvatum by insertion of a straight nail in both antegrade and retrograde femoral lengthening.

The 2 patients with preoperative procurvatum deformity were excluded from this calculation. 1 patient developed an unintended procurvatum deformity of 7° during the course of lengthening.

#### Frontal and sagittal plane tibia

In the tibia, no change of MTPA could be observed in the 6 patients included in the current study; however, 3 patients showed reduction in PPTA (3°, 3°, and 9°) (Figure 3).

**Figure 1:**
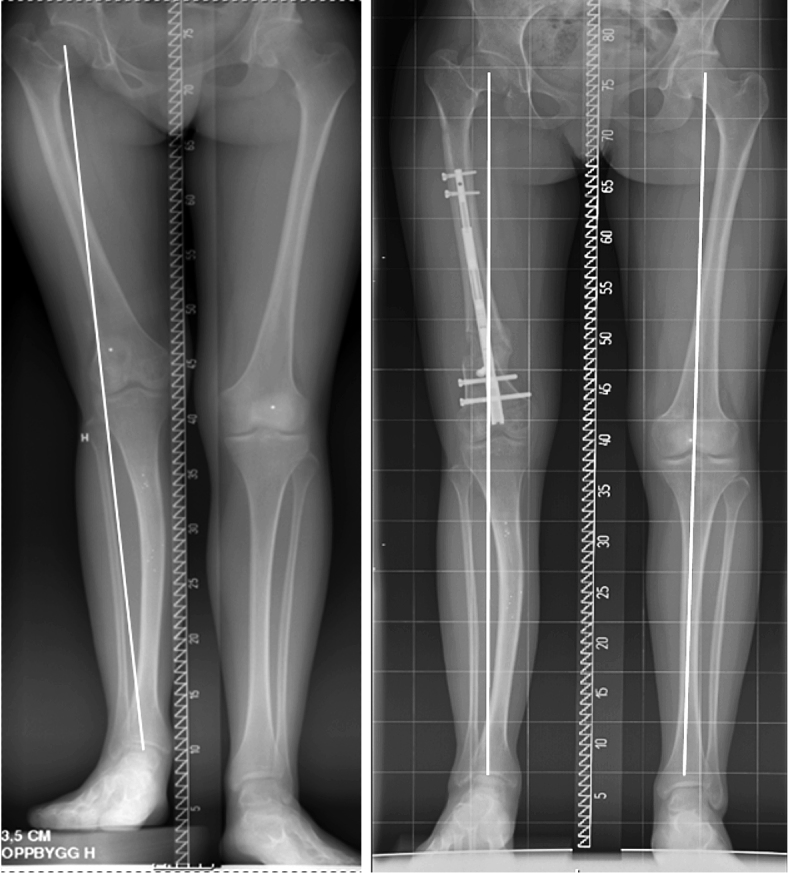
20-year-old woman with CFD and fibular hemimelia. Initial shortening and valgus in the femur and valgus in the tibia. Femoral valgus and shortening were corrected with the retrograde nail technique; some remaining knee valgus due to deformity in the tibia. However, acceptable mechanical axis and currently no further surgery required. High-riding trochanter, but no Trendelenburg gait.

**Figure 2:**
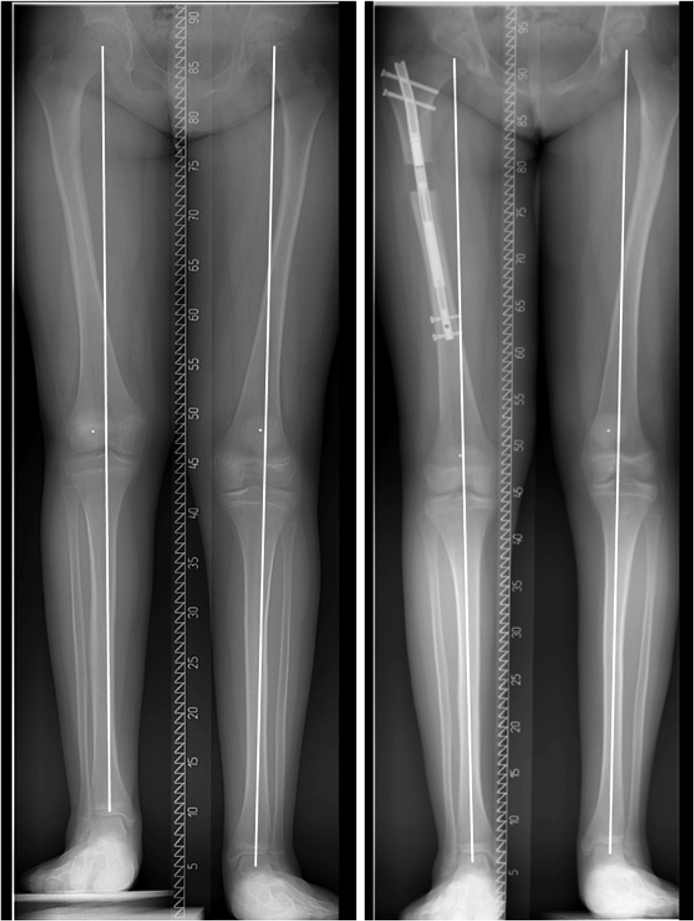
11-year-old girl with cerebral paresis and hemiplegia on her left side. Initial leg length discrepancy was 3 cm, where the hemiplegic side was the longer extremity. She had serious gait problems and gait analysis with shoe augmentation of 4 cm on the right side showed significant improvement of gait parameters. The girl was lengthened 4 cm in her right femur, overcorrecting her by 1 cm, which was advantageous with respect to her left-sided hemiplegia and future growth. Long standing radiographs demonstrate that no shift of MA axis was observed.

**Figure 3:**
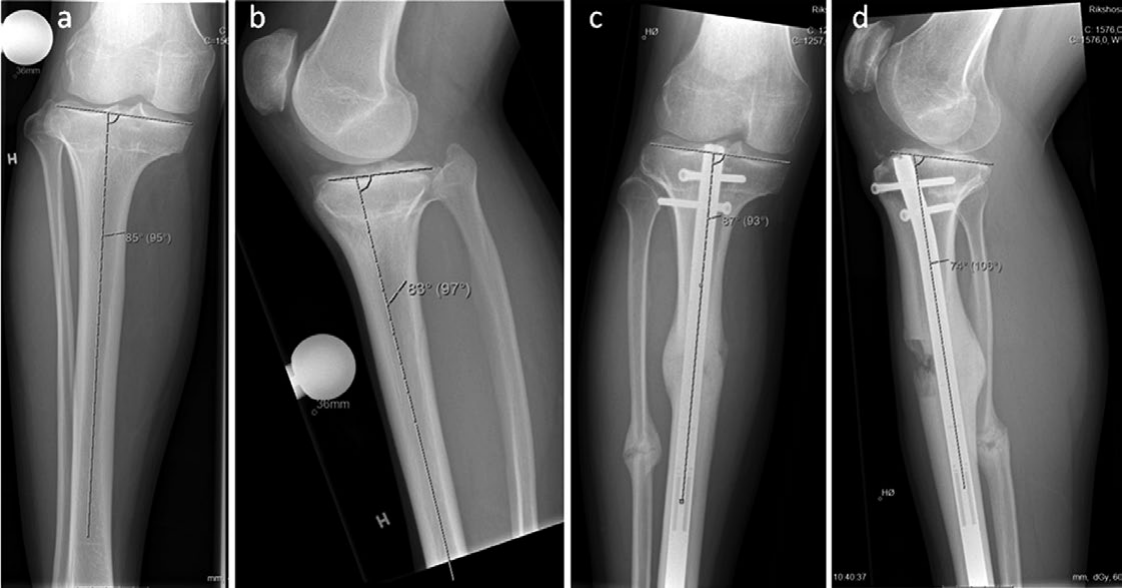
30-year-old man with 35 mm of tibial shortening due to traumatic injury to the proximal tibial growth plate in childhood. Furthermore, the patient had a symptomatic high-riding fibular head (a). Initial PPTA was slightly below normal (b). The patient was lengthened with a tibia Precice® nail. The fibula was osteomized, but transfixation was done only between the tibia and fibula distally to the osteotomy. This resulted in some lengthening of the fibula as well as an intended distalization of the fibular head (c). However, PPTA increased slightly from preoperatively, which was not intended (d). Delayed healing of the regenerate anteriorly (d). However, there was solid callus on 3 cortices (c, d).

### Healing of the regenerate

**Figure 4:**
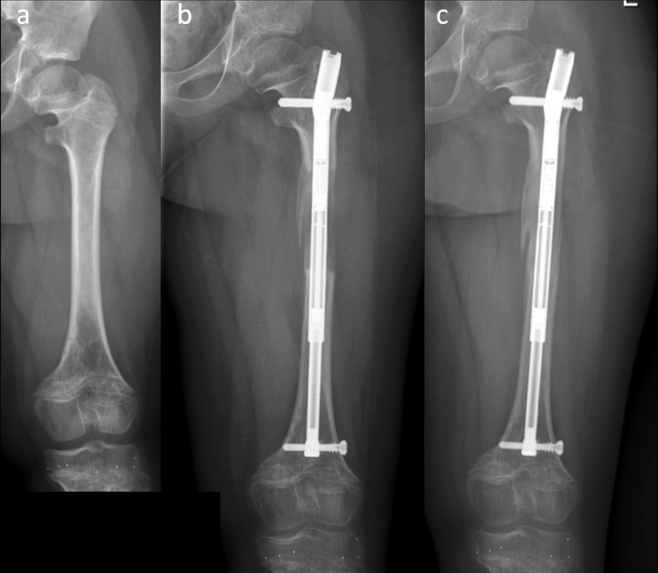
A 12-year-old girl with achondroplasia. She underwent consecutive 50 mm lengthenings of both femurs with the shortest available Precice nail (16.5 cm). Radiographs preoperatively (a), when lengthening was completed (b) and after consolidation (c). The nail has only one locking option proximally and distally to allow for 50 mm lengthening, despite the shortness of the nail. The patient’s femurs had not been lengthened earlier. Lengthening indices for both femurs were 0.6 months/cm, the fastest healing of all procedures included in the current study.

All but 1 lengthening consolidated without further interventions. In the femur the lengthening index was mean 1.2 months/cm (0.6–2.5), whereas the lengthening index for the AFN was 1.1 months/cm (0.6–2.0) and 1.3 months/cm (0.8–2.5) for the RFN (Figure 4). Differences in lengthening indices between the AFN and RFN were statistically significant (p = 0.03). However, in 15 patients receiving RFN an axial correction was performed when the nail was inserted. Exclusion of these patients and comparison of isolated lengthening procedures with the RFN and AFN did not show any statistically significant difference in lengthening indices between the two techniques (p = 0.2). The lengthening index in the tibia was 2.5 months/cm (1.6–4.0). Mean lengthening indices in the femur were for children (≤ 18 years) 0.9 months/cm (0.6–1.3), and 1.4 months/cm (0.8–2.5) for adults. This difference was statistically significant (p = 0.005).

### Precision of MA analysis

As a routine, long standing anterior-posterior radiographs of all patients were obtained before surgery and after healing of the regenerate. These radiographs were taken of both lower extremities simultaneously. In those 28 patients with a healthy contralateral extremity and available radiographs from 2 different time points, a calculation of the precision of our long standing radiograph measurements could be done. MAD at the first measurement was mean 1 mm (–20 to 16) and –1 mm (–19 to 19) at the second measurement. The mean change of MAD between the 2 measurements was 3 mm (0–7). Precision of the MA measurements on our long standing radiographs was therefore assumed to be ±3 mm.

### Complications

**Figure 5:**
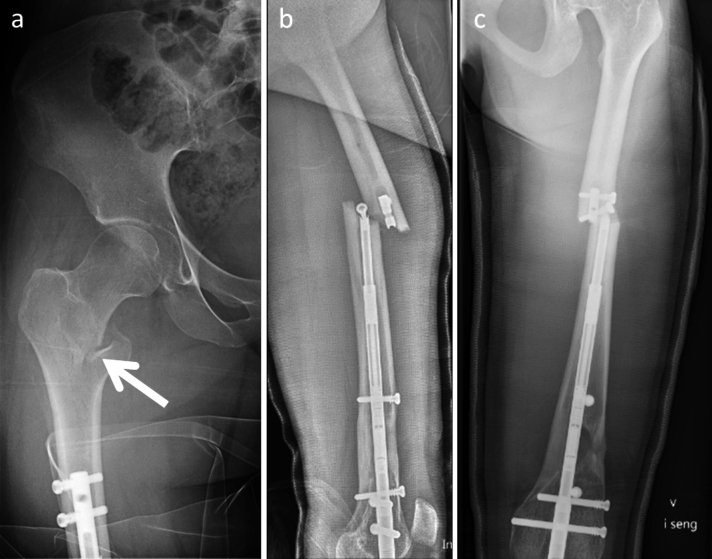
A 24-year-old woman who was lengthened 30 mm for idiopathic LLD with a retrograde lengthening nail. After consolidation of the regenerate she fell from a bicycle, sustaining a pertrochanteric femoral fracture (a). A 16-year-old girl with CFD, who underwent 40 mm of lengthening and correction of a valgus deformity with a retrograde lengthening nail. After consolidation of the regenerate she fell 2 m in a waterfall, sustaining a femoral fracture and breakage of the nail at the level of a locking bolt (b).

8 complications occurred that could be solved by surgery without sequelae, and were therefore graded as obstacles according to Paley (1990): in 1 patient (amniotic band syndrome, 40 mm tibial lengthening) autologous bone grafting was required to achieve healing, which occurred within 3 months after that procedure. In 1 patient, an AFN had to be exchanged due to failure of the lengthening mechanism (Precice nail), which was considered to be caused by too extensive hammering during insertion of the nail. 3 patients had to be revised due to migration of locking screws, and 1 patient due to insufficient connection of the receiver in a Fitbone nail. 2 patients sustained femoral fractures due to adequate trauma (fall from bicycle and an accident in a waterfall) 2 and 3 months after consolidation of the regenerate and initiation of full weight-bearing (Figure 5). Both patients were lengthened with RFN, which had not been removed at the time of fracture. Both fractures were treated with open reduction and osteosynthesis with locking compression plates. There were no postoperative infections, and no other problems, obstacles, or complications occurred.

## Discussion

Controlled axial correction and lengthening could be achieved with an externally controlled motorized lengthening nail, which confirms findings by other authors (Krieg et al. 2011, Al-Sayyad 2012, Fragomen and Rozbruch 2017). Residual minor deformity was present in one-third of our cases where acute axial corrections were performed during nail insertion. To avoid this, thorough preoperative planning and intraoperative control of alignment are required. Furthermore, unintentionally induced minor frontal plane deformities occurred in one-third of the antegrade femoral lengthenings, resulting in a substantial shift of the MA in 1 patient. It is essential to insert the nail at the very tip of the trochanter to reduce the risk of varization of fragments, and blocking screws might be used to control alignment (Baumgart 2009, Muthusamy et al. 2016, Hammouda et al. 2017). Trochanteric entry can be used in femoral nailing in the skeletally immature patient (MacNeil et al. 2011, Hammouda et al. 2017). We used trochanteric entry point in all patients with an AFN. A fossa piriformis entry point might be less frequently associated with varization of fragments and can be safely used in adult patients (Kim et al. 2016).

Unintentionally induced minor deformity in the sagittal plane in femoral lengthening could be observed in most patients and is caused by the fact that a straight nail is inserted into a naturally flexed femur. Adverse clinical effects on range of motion and function have not been observed.

In cases of isolated lengthening no shift of mechanical axis to the lateral could be observed, although lengthening with nails is performed along the anatomical axis of the femur. This might be due to 2 reasons: (1) the amount of lengthening was not great enough to result in a measurable shift of MA, detectable on long standing radiographs, considering the accuracy of this measurement of ±3 mm; (2) in more than one-third of the procedures some unintended varization of the proximal fragment in antegrade nailing was induced, which may have compensated for the effect of lengthening along the anatomical axis to some degree.

Few tibial lengthenings were included in our study. Most frequently, unintended procurvatum and valgus deformity can occur with tibial lengthenings (Muthusamy et al. 2016). However, we only observed minor unintended procurvatum deformity of no clinical significance in our study.

All femurs healed within an appropriate time, and most of them showed fast healing, which corresponds to findings by other authors (Krieg et al. 2011, Kucukkaya et al. 2015, Karakoyun et al. 2016, Laubscher et al. 2016). Healing in the femur seems faster, when the patient’s age is ≤18 years, which might have importance for the timing of reconstructive procedures. In the tibia slower healing was observed. However, the number of tibial lengthenings was low and all patients who underwent tibial lengthening had an underlying diagnosis associated with reduced bone healing potential.

The number of complications was low (8/50), although additional surgery was required in these patients. However, the current study analyses all our first 50 consecutive cases and the method certainly has a learning curve allowing for reduction of the number of complications with increasing experience. 2 patients sustained a femoral fracture due to trauma after consolidation of the regenerate and return to full weight-bearing. Besides the occurrence of these 2 femoral fractures we found similar outcome measures between the antegrade and retrograde nailing technique in the femur and there are no other reports comparing these 2 lengthening techniques. Nevertheless, we prefer the antegrade approach in the femur when isolated lengthening is performed. A strength of our study is that we report all consecutive lengthenings with externally controlled intramedullary nails that have been performed at our department.

In summary, both femoral and tibial motorized lengthening nails are reliable implants for limb lengthening, with a low number of complications. Preoperative planning, intraoperative control, and adequate postoperative care and follow-up are essential in order to achieve a good result.

JH, SH, IH, and AB performed the surgeries and examined the patients at follow-up. JH wrote the manuscript. HS and IH revised the manuscript.

*Acta* thanks John Gerard Birch and Hubert Jan Oostenbroek for help with peer review of this study.
